# Discovery of Herbal Remedies and Key Components for Major Depressive Disorder Through Biased Random Walk Analysis on a Multiscale Network

**DOI:** 10.3390/ijms26052162

**Published:** 2025-02-28

**Authors:** Jun-Ho Lee, Sungyoul Choi, Do-Eun Lee, Hyung Won Kang, Jin-Seok Lee, Ji-Hwan Kim

**Affiliations:** 1 Department of Oriental Pharmacy, College of Pharmacy, Wonkwang University, Iksan 54538, Republic of Korea; 2 College of Korean Medicine, Gachon University, Seongnam 13120, Republic of Korea; 3 Department of Korean Neuropsychiatry Medicine, College of Korean Medicine, Wonkwang University, Iksan 54538, Republic of Korea; 4 Institute of Bioscience & Integrative Medicine, Daejeon Hospital of Daejeon University, Daejeon 35235, Republic of Korea; 5 Department of Sasang Constitutional Medicine, Division of Clinical Medicine, School of Korean Medicine, Pusan National University, Yangsan 50612, Republic of Korea

**Keywords:** major depressive disorder, herbal medicine, active components, multiscale network

## Abstract

Major depressive disorder (MDD) is a widespread psychiatric condition with substantial socioeconomic impacts, yet single-target pharmacotherapies often yield responses. To address its multifactorial nature, this study employed a multiscale network analysis of herbs, their active components, and MDD-associated protein targets. Using a biased random walk with restart, we calculated interactions between disease-related and herb-derived targets, identifying herbs highly correlated with MDD. Enrichment analysis further revealed key signaling pathways, including oxidative stress, neuroinflammation, and hormone metabolism, underlying these herbs’ therapeutic effects. We identified *Ephedrae herba*, *Glehniae radix*, *Euryales semen*, and *Campsitis flos* as promising candidates, each containing multiple bioactive compounds (such as ephedrine, psoralen, xanthine, and ursolic acid) that modulate critical processes like oxidation–reduction, inflammatory cytokine regulation, and transcriptional control. Network visualization showed how these herbs collectively target both shared and distinct pathways, supporting a synergistic, multi-target therapeutic strategy. This approach underscores the significance of network-based methodologies in addressing complex disorders such as MDD, where focusing on a single target may overlook synergistic interactions. By integrating diverse molecular data, this study provides a systematic framework for identifying novel interventions. Future experimental validation will be crucial to confirm these predictions and facilitate the translation of findings into effective MDD therapies.

## 1. Introduction

Major depressive disorder (MDD) is the most prevalent neuropsychiatric disorder worldwide, significantly reducing quality of life and imposing a substantial economic burden on society. In the United States alone, the total cost of MDD soared from approximately USD 23.6 billion in 2010 to USD 33.37 billion by 2019, including USD 960 million in losses due to premature mortality. Notably, suicides attributed to MDD account for roughly one-third of all suicide cases in the country, underscoring its impact on public health [1]. This burden has only intensified in the wake of the COVID-19 pandemic, with MDD prevalence estimated to have risen by around 25% during the first year of the crisis [2]. Recognized as a public health and societal challenge, MDD is driven by complex etiological factors—including neurotransmitter imbalances, genetic predispositions, environmental stressors, inflammatory processes, and gut–brain axis dysregulation—that collectively hinder effective management [3,4]. Given the clinical and economic impact of MDD, a comprehensive understanding of its underlying mechanisms is crucial for developing more effective treatment strategies.

Current treatment options for MDD—including selective serotonin reuptake inhibitors (SSRIs), serotonin–norepinephrine reuptake inhibitors (SNRIs), and tricyclic antidepressants (TCAs)—are widely recognized as first-line therapies [5,6,7]. However, up to one-third of patients do not achieve remission despite adequate trials, resulting in treatment-resistant depression (TRD) [8]. To address TRD, clinicians often resort to augmentation or combination strategies and, in some cases, novel agents such as esketamine nasal spray (Spravato), approved for use in TRD. It modulates the NMDA receptor, offering a relatively rapid antidepressant effect compared to traditional monoamine-based treatments [9]. Nonetheless, it is not without limitations: potential adverse effects—including dissociation, sedation, and the risk of abuse—necessitate strict monitoring and restricted administration settings. Furthermore, conventional antidepressants commonly cause side effects such as gastrointestinal disturbances, sleep disorders, and weight gain, which can hamper treatment adherence and reduce overall effectiveness. Given these challenges, there is a need to develop safer and more efficacious therapeutic strategies to better address the diverse and complex needs of MDD patients.

There has been growing interest in multi-target approaches to overcome the limitations of traditional single-target strategies. Network pharmacology, which integrates and analyzes the interactions between disease-related genes or proteins and drugs or herbal components from a network perspective, has emerged as a promising method for elucidating the mechanisms of complex diseases and identifying new therapeutic candidates [10]. Notably, herbal medicines possess inherent “multi-component, multi-target” characteristics, making them particularly suitable for the development of treatments for multifactorial diseases such as MDD. Unlike conventional synthetic drugs that typically act on a single target, herbal medicines contain multiple bioactive compounds capable of simultaneously modulating diverse physiological systems—potentially yielding synergistic effects [11]. Furthermore, their long-standing use in traditional medicine suggests generally favorable safety profiles, supporting their promise as an improved treatment option for MDD.

**Figure 1:**
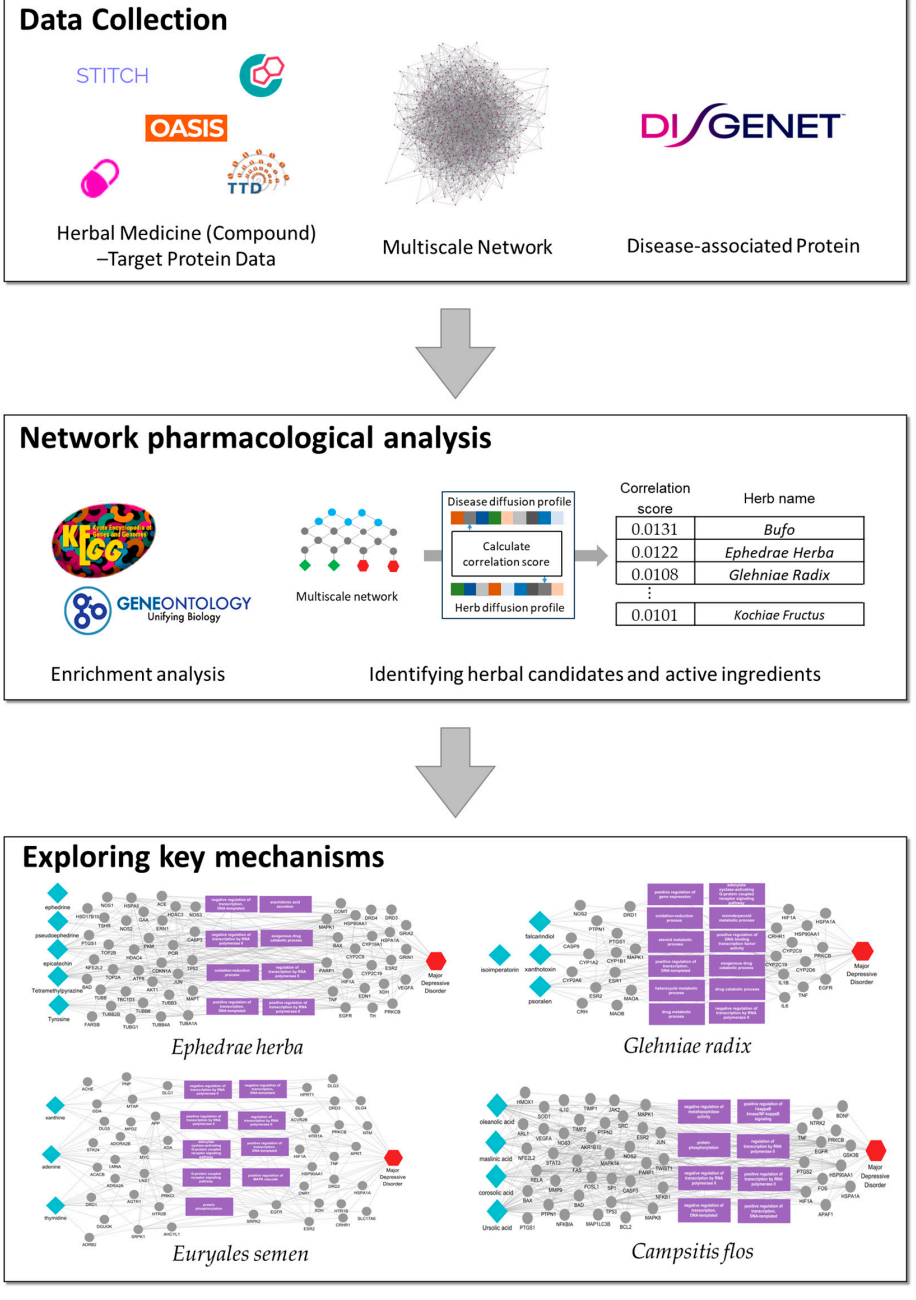
Schematics for identifying candidate herbs and active compounds for MDD. This schematic depicts the application of multiscale network analysis to identify herbs and their active compounds with potential efficacy against MDD. Herb–compound and compound–target interactions were mapped, followed by the identification of disease-associated proteins. Diffusion profiles for the herbs and disease-related proteins were computed and compared, leading to the prioritization of herbs based on their correlation scores. Enrichment analysis was performed to uncover pivotal biological pathways, while individual active components of the top-ranked herbs were analyzed to identify key protein targets. The lower panel illustrates the key mechanism of the selected herbs against MDD by visualizing edges between the herbs’ active components and the disease, with proteins and biological functions as intermediaries.

In this study, we employed a multiscale network analysis to identify candidate herbal medicines and their active compounds for MDD (Figure 1). Specifically, we constructed an MDD-centric herb–compound–target–biological function network and then applied a biased random walker algorithm to simulate disease and herb impacts within this network. Based on these simulations, we prioritized candidate herbs and investigated their biological functions and key signaling pathways. Among the prioritized herbs, we selected one with no previously reported evidence in the context of MDD and predicted its active compounds. Finally, we explored the critical mechanisms of these compounds in the multiscale disease network. Taken together, these findings not only highlight the value of integrative approaches for unraveling the complex pathophysiology of MDD but also provide a systematic framework for discovering effective interventions.

## 2. Results

### 2.1. Screening Candidate Herbs for Major Depressive Disorder Treatment

To identify herbal medicines with potential efficacy for major depressive disorder (MDD), we initially collected herbal medicine–compound data from the OASIS database. We then extracted the corresponding target proteins of these compounds from validated databases such as DrugBank, TTD, and STITCH. Using the target data from these herbal medicines, MDD-related targets, and the proteins and biological functions embedded within the multiscale network, we applied a biased random walk algorithm to perform diffusion profiling. Based on these diffusion profiles, we computed correlation scores that represent the associations between each herbal medicine and MDD, selecting those with high scores as promising candidate herbs for MDD treatment. In addition, a hypergeometric test was conducted to assess the degree of protein overlap. From these analyses, we identified herbal medicines that exhibited both high correlation scores and statistically significant relationships (*p*-value < 0.05) with disease-related proteins (Table 1).

The overlap enrichment value between MDD-related target proteins and the candidate herbal medicines averaged 4.75, confirming that the multiscale network-based prediction model effectively identified targets closely associated with MDD. Analysis revealed that Bufo showed the highest correlation score (0.01312), followed by *Ephedrae herba* (0.01219), *Glehniae radix* (0.01078), *Crotonis semen* (0.01056), *Euryales semen* (0.01038), *Acori graminei rhizoma* (0.01038), and *Campsitis flos* (0.01034). Among the top 10 selected herbs, *Bufo*, *Crotonis semen*, and *Acori graminei rhizoma* have previously been reported to have the potential to exhibit beneficial effects on MDD [12,13,14,15,16], demonstrating that our predictive results are consistent with existing experimental evidence. In contrast, *Ephedrae herba*, *Glehniae radix*, *Euryales semen*, and *Campsitis* have seen little to no prior research for MDD treatment. Nonetheless, in this study, they showed above-average enrichment values and significant protein overlap with the MDD profile. These findings suggest that these candidate herbs may serve as promising novel therapeutic agents for MDD.

### 2.2. Herb–Compound–Target Network Construction Focused on Prioritized Herbs

**Figure 2:**
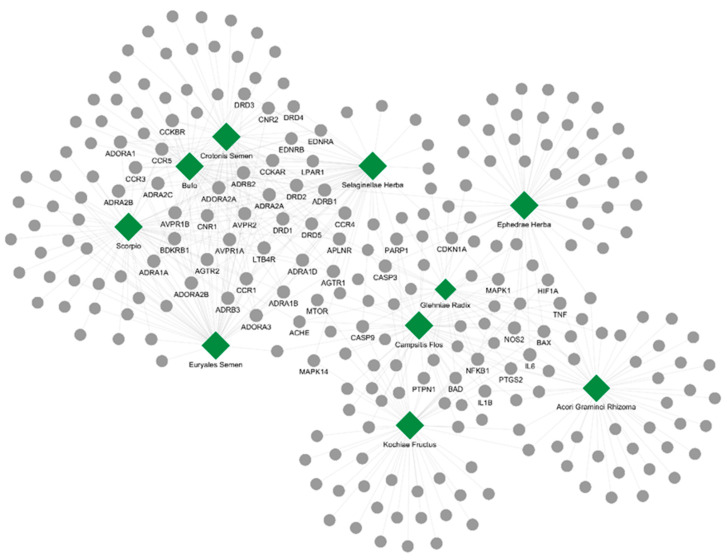
Herb–target interaction network of the top 10 candidate herbs with high correlation scores for MDD. Green hexagons represent herbs, and gray circles represent protein targets. Edges indicate interactions between herbs and targets, with the size of hexagons and circles reflecting interaction frequency.

We constructed a network consisting of the interactions between the top 10 herbal medicines—those displaying the highest correlation scores with MDD—and their corresponding target proteins (Figure 2). This network comprised a total of 477 interactions (edges) among the 10 herbal medicines and 250 targets, effectively illustrating how multi-target characteristics and complex biological interactions inherent in multi-herbal formulations can lead to potential therapeutic effects. Of these targets, only 55 proteins were co-targeted by three or more herbal medicines, suggesting that they may serve as key targets. Notably, the neurotransmitter receptors ADORA2B, ADORA1, and ADORA2A showed the highest cumulative frequencies, followed closely by inflammation-related proteins such as NFKB1, IL6, IL1B, and TNF. These findings indicate that these key target proteins likely play critical roles in the mechanisms of herbal medicines and may serve as pivotal targets in the treatment of MDD.

### 2.3. Enrichment Analysis of Prioritized Herbs

Enrichment analysis using both KEGG and Gene Ontology was conducted to further explore the key target proteins of the herbs. Our goal was to identify the signaling pathways and biological functions linked to the top 10 chosen herbs. The KEGG results revealed significant associations with several key pathways, such as the neuroactive ligand–receptor interaction pathway, calcium signaling pathway, and cGMP-PKG signaling pathway. We also found that some pathways are associated with inflammatory cytokine activation, like the TNF and IL-17 signaling pathways (Table 2).

**Figure 3:**
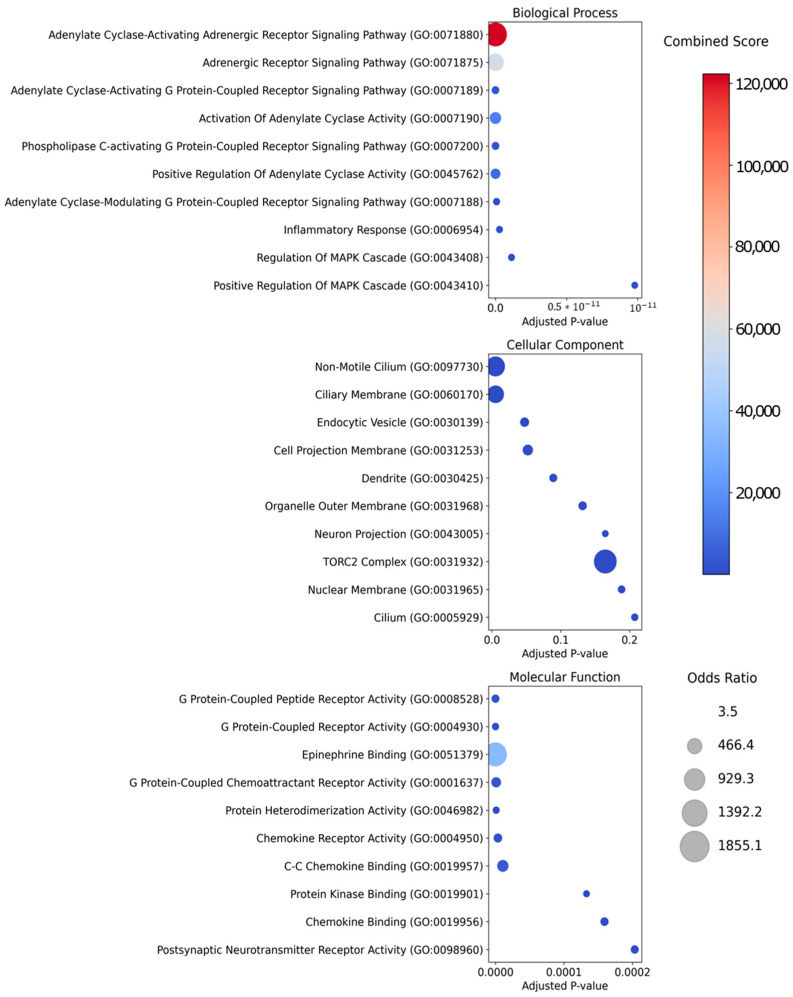
Gene Ontology enrichment analysis for core protein targets. A Gene Ontology enrichment analysis was conducted on 55 key protein targets, categorized into three domains: biological processes (**top**), cellular components (**middle**), and molecular functions (**bottom**). The *x*-axis displays the adjusted *p*-value, representing the significance of the associations, while the bubble size indicates the odds ratio. The bubble color reveals the combined score, which indicates the statistical significance of each term. This visualization emphasizes the most significantly enriched terms within each category.

Gene Ontology analysis revealed strong associations with key biological processes centered around adrenergic and G protein-coupled receptor signaling pathways, notably those involving the activation and positive regulation of adenylate cyclase (Figure 3, top). Additional significant links were observed in the inflammatory response and MAPK cascade regulation, underscoring the potential role of these key targets in regulating immune and intracellular signaling processes. Examination of the cellular components (Figure 3, middle) showed these targets to be predominantly localized in cilia, endocytic vesicles, dendrites, and other membrane-bound structures—organelles crucial for cellular communication, transport, and signal transduction. Molecular function analysis (Figure 3, bottom) indicated high combined scores for G protein-coupled receptor activity, epinephrine binding, and chemokine receptor activity. These results suggest that the key targets are heavily involved in adrenergic signaling and immunological processes. Taken together, these findings highlight the central importance of these core protein targets in regulating multiple signaling pathways.

### 2.4. Identification of Potential Active Compounds and Multiscale Network Analysis of Prioritized Herbs

We performed an additional analysis of the active compounds and multiscale network mechanisms of the prioritized herbs *Ephedrae herba*, *Glehniae radix*, *Euryales semen*, and *Campsitis flos*, identified as potential treatments for MDD. To assess the potential impact of each herb’s active compounds on MDD, correlation scores were computed between the diffusion profiles of each herb’s active compounds and MDD-related proteins. Based on these scores, up to five active compounds were selected for further analysis. A multiscale network was then constructed by integrating target protein data for both the selected compounds and MDD-related proteins, along with data on their relevant biological functions. The most influential proteins and biological processes in both the herbal compounds and MDD-related mechanisms were subsequently extracted and visualized.

**Figure 4:**
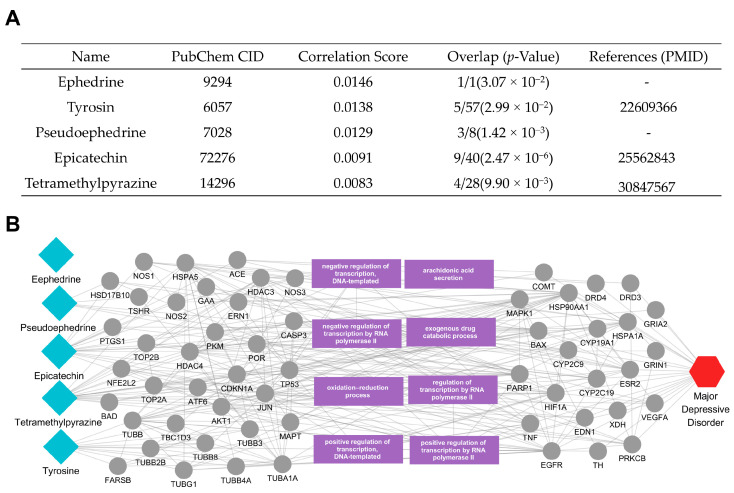
Active compounds of *Ephedrae herba* and their association with MDD. (**A**) The table lists five active compounds—ephedrine [17], pseudoephedrine, epicatechin [18], tetramethylpyrazine [19], and tyrosine—along with each compound’s correlation score and protein overlap *p*-value in relation to MDD. (**B**) The network diagram illustrates how these compounds (blue diamond nodes, left) connect to their target proteins and the relevant biological processes (gray and purple, center), ultimately linking to the MDD node (red hexagon, right). Edges represent interactions or functional relationships, highlighting potential mechanisms through which *Ephedrae herba* may modulate MDD pathophysiology.

Focusing first on *Ephedrae herba*, we analyzed its active compounds and the relationships they form with their target proteins (Figure 4A). Ephedrine and pseudoephedrine were found to connect to several MDD-related targets, including NOS2, TNF, and NFKBIA (Figure 4B). NOS2 is involved in the oxidation–reduction process and contributes to nitric oxide production—an important mediator in vascular regulation and neuroinflammation. Additionally, TP53 and NFE2L2 are key regulators of oxidative stress responses, playing central roles in cellular homeostasis and damage repair. Another critical function highlighted in the network is the regulation of transcription by RNA polymerase II, with HDAC4 and ATF6 emerging as pivotal transcription modulators that influence stress responses, protein folding, and metabolic pathways. Furthermore, NFKBIA negatively regulates NF-κB transcriptional activity, thereby modulating inflammatory signaling and immune responses.

**Figure 5:**
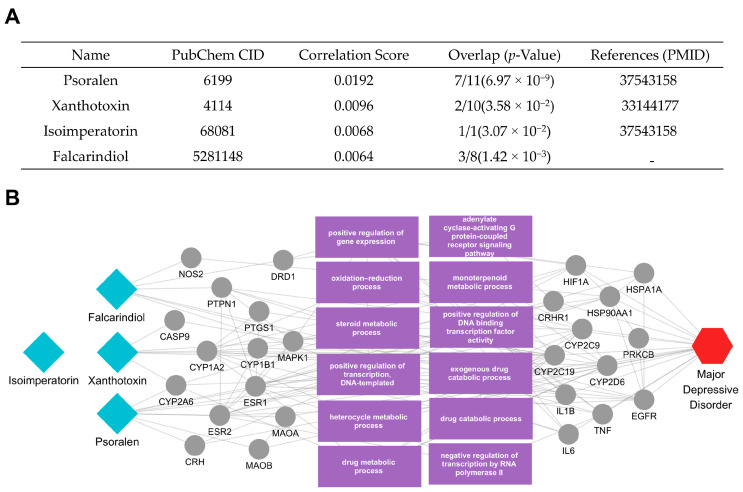
Active compounds of *Glehniae radix* and their association with MDD. (**A**) The table lists four active compounds—psoralen [20], xanthotoxin [21], isoimperatorin [22], and falcarindiol—along with each compound’s correlation score and protein overlap *p*-value in relation to MDD. (**B**) The network diagram reveals how these compounds (blue diamond nodes, left) connect to their target proteins and the relevant biological processes (gray and purple, center), ultimately linking to the MDD node (red hexagon, right). Edges represent interactions or functional relationships, highlighting potential mechanisms through which *Glehniae radix* may modulate MDD pathophysiology.

Next, we conducted an analysis of the active compounds and key mechanisms of *Glehniae radix*. For this analysis, we selected four compounds—isoimperatorin, xanthotoxin, falcarindiol, and psoralen—prioritizing their correlation scores and *p*-values (Figure 5A). The key elements connecting these compounds to MDD were extracted, and their relationships were visualized (Figure 5B). The analysis revealed that isoimperatorin was directly linked to the MDD-related proteins DRD1 and NOS2, while xanthotoxin interacted with ESR1, MAOA, and ESR2. Additionally, falcarindiol targeted proteins such as PTGS1, CASP9, and PTPN1, whereas psoralen modulated CYP2A6 and CRH. These targets were shown to be involved in diverse interaction networks tied to neuroinflammation, stress responses, and neurotransmitter regulation.

Further investigation highlighted several biological functions relevant to MDD pathophysiology, including the oxidation–reduction process and the steroid metabolic process. In particular, the oxidation–reduction process plays a crucial role in managing cellular redox balance and mitigating oxidative stress—an aspect increasingly implicated in depressive symptomatology. Meanwhile, the steroid metabolic process encompasses pathways involved in hormone synthesis and regulation, affecting neuroendocrine function and stress reactivity.

**Figure 6:**
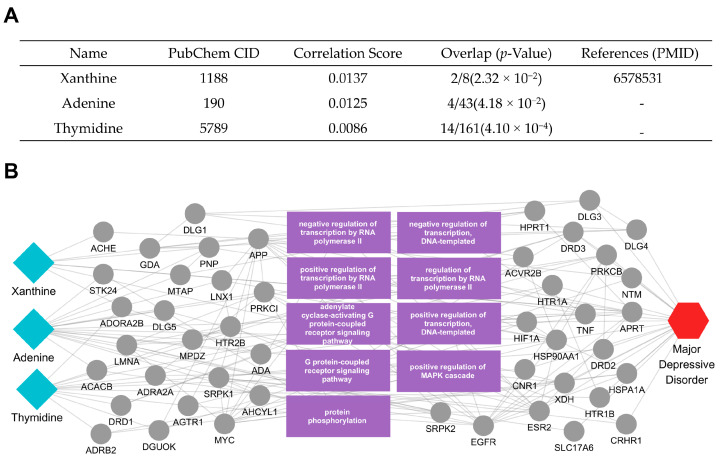
Active compounds of *Euryales semen* and their association with MDD. (**A**) The table lists three active compounds—xanthine [23], adenine, and thymidine—along with each compound’s correlation score and protein overlap *p*-value in relation to MDD. (**B**) The network diagram reveals how these compounds (blue diamond nodes, left) connect to their target proteins and the relevant biological processes (gray and purple, center), ultimately linking to the MDD node (red hexagon, right). Edges represent interactions or functional relationships, highlighting potential mechanisms through which *Euryales Semen* may modulate MDD pathophysiology.

Similarly, we examined the active compounds and mechanisms of *Euryales semen*, focusing on xanthine, adenine, and thymidine (Figure 6A). After constructing a multi-layer network, we identified the key elements most pertinent to MDD and visualized their interrelationships (Figure 6B). Our analysis revealed that xanthine was linked to MDD-related proteins such as ACHE, GDA, and PNP, whereas adenine modulated targets including ADRA2A, MYC, and STK24. Meanwhile, thymidine interacted with DRD1 and ADRB2, both of which are pivotal for neurotransmitter signaling and stress responses.

Notably, these proteins are tied to critical biological processes highlighted in the network—such as negative and positive regulation of transcription (by RNA polymerase II or DNA-templated), adenylate cyclase-activating G protein-coupled receptor signaling, G protein-coupled receptor signaling, positive regulation of the MAPK cascade, and protein phosphorylation. Each of these functions plays a significant role in MDD pathophysiology, influencing monoamine release, stress hormone regulation, and cytokine production. By modulating such a diverse array of pathways, the active compounds of *Euryales semen* may collectively help mitigate depressive symptoms through multifaceted mechanisms of action. 

**Figure 7:**
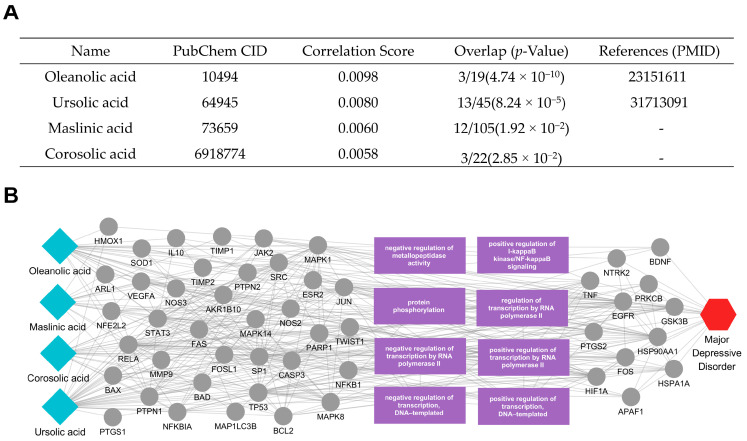
Active compounds of *Campsitis flos* and their association with MDD. (**A**) The table lists four active compounds—oleanolic acid [24], maslinic acid, corosolic acid, and ursolic acid [25]—along with each compound’s correlation score and protein overlap *p*-value in relation to MDD. (**B**) The network diagram reveals how these compounds (blue diamond nodes, left) connect to their target proteins and the relevant biological processes (gray and purple, center), ultimately linking to the MDD node (red hexagon, right). Edges represent interactions or functional relationships, highlighting potential mechanisms through which *Campsitis flos* may modulate MDD pathophysiology.

We conducted an additional analysis of the active compounds and key mechanisms of *Campsitis flos* (Figure 7A). Based on the compound analysis results, we focused on four top-ranking triterpenic acids—oleanolic acid, maslinic acid, corosolic acid, and ursolic acid—which exhibited high correlation scores and significant protein overlap, suggesting potential contributions to MDD (Figure 7B). These compounds were found to target proteins such as HMOX1, NFE2L2, MAPK14, IL10, SOD1, and JAK2, each of which is directly associated with MDD-related factors including TNF, BDNF, GSK3B, and HIF1A. Notably, additional elements like NFKBIA, CASP3, FOSL1, and REL emerged as critical regulators of MDD-associated pathways.

Many of these targets participate in negative regulation of metallopeptidase activity, positive regulation of I-kappaB kinase/NF-kappaB signaling, protein phosphorylation, and various modes of transcriptional regulation (both RNA polymerase II- and DNA-templated), as indicated by the purple-labeled functions in the network. Collectively, these processes influence inflammatory signaling, anti-oxidative defense (e.g., via HMOX1 and SOD1), and stress-induced apoptosis (e.g., via CASP3 and MAPK pathways)—all key factors in MDD pathophysiology. By modulating cytokine levels, neuroprotective signaling, and gene transcription, the triterpenic acids in *Campsitis flos* may help restore homeostatic balance in multiple pathways implicated in depression, underscoring their potential therapeutic relevance.

### 2.5. Reliability Assessment of Predicted Targets via Comparison with Existing Experimental Data

**Figure 8:**
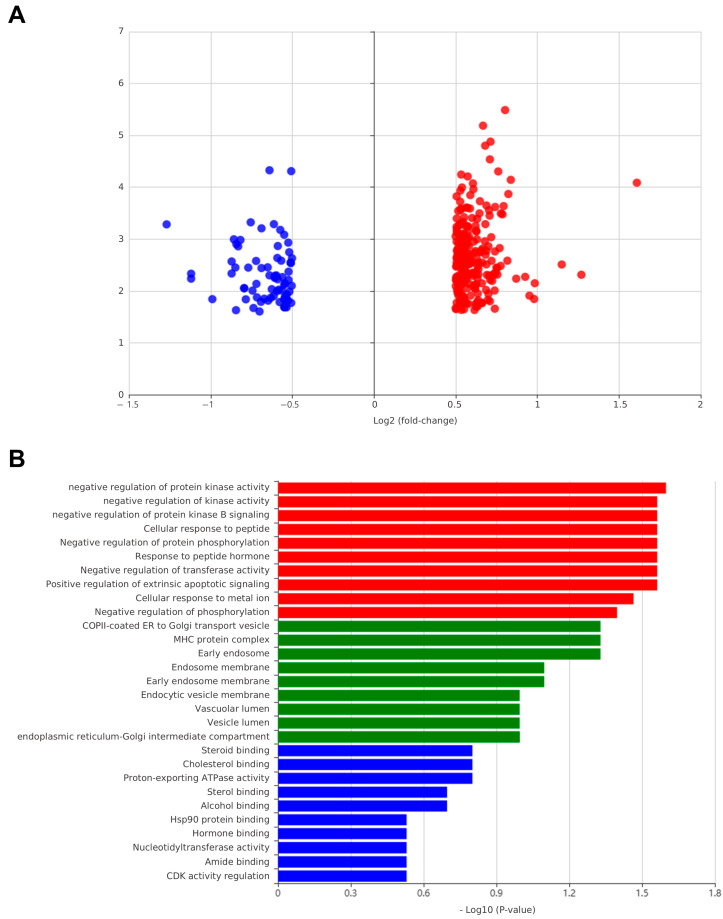
Transcriptomic profile and GO enrichment of oleanolic acid-related genes. (**A**) Volcano plot illustrating log2 (fold-change) in gene expression between upregulated (red) and downregulated (blue) transcripts following treatment with oleanolic acid. The *y*-axis represents −log10(adjusted *p*-value), highlighting the most statistically significant differentially expressed genes (DEGs). (**B**) Gene Ontology (GO) enrichment analysis of the DEGs, grouped by domain: biological processes (red), cellular components (green), and molecular functions (blue).

We further evaluated the reliability of our computational predictions by investigating the transcriptome-wide effects of oleanolic acid, a prioritized active compound in Campsitis Flos. Using HERB and CMap analyses, we first identified compounds with a connectivity score of 80 or higher and found that several, including sertraline, memantine, parthenolide, and celastrol, showed gene expression profiles similar to that of oleic acid (Supplementary Table S1). In addition, an examination of transcriptomic changes following oleic acid treatment revealed that 227 genes were upregulated and 72 genes were downregulated (Figure 8A). Further gene-set enrichment analysis suggested that oleic acid might regulate biological processes such as in the negative regulation of protein kinase activity, the response to peptide hormones, and hsp90 protein binding (Figure 8B). These findings imply a potential impact of oleanolic acid on these pathways. 

## 3. Discussion

Major depressive disorder (MDD) is a complex and often treatment-resistant condition, emphasizing the need for alternative therapeutic approaches. In this study, we employed a multiscale network pharmacology approach to identify and prioritize candidate herbal medicines for MDD. By integrating data on herbal medicines, their active compounds, and disease-associated targets, we constructed an MDD-focused herb–compound–target–biological function network and applied a biased random walk. These methods allowed us to rank the herbs based on their correlation with MDD-related proteins. Our analysis not only confirmed previously recognized herbs with antidepressant effects (e.g., *Bufo*, *Crotonis Semen*) but also revealed several novel candidates, including *Ephedrae herba*, *Glehniae radix*, *Euryales semen*, and *Campsitis flos*, which exhibited significant correlations and enrichment for MDD-associated targets. Furthermore, enrichment analysis results highlighted key pathways—such as those involved in neurotransmitter signaling, inflammation, and stress responses—offering mechanistic insights into how these herbs may modulate MDD pathophysiology. Overall, these findings underscore the potential utility of an integrative approach in discovering therapeutic strategies for psychiatric disorders like MDD.

A key strength of our method lies in its ability to integrate multiple layers of biological information—ranging from molecular targets to higher-order biological functions—within a cohesive framework. Whereas conventional approaches often focus on a single pathway or molecule, our multiscale network-based strategy can capture the complex interplay among diverse factors underlying MDD. Indeed, previous studies have demonstrated that the multi-layer network analysis method, which identifies active compounds and key mechanisms by computing and comparing diffusion profiles, is more effective than traditional research approaches [26,27]. By applying a biased random walk algorithm to quantify similarities in diffusion profiles between disease proteins and herb targets, we not only reveal potential synergies among active compounds but also uncover latent functional connections. This comprehensively enables the identification of critical nodes where multiple pathogenic mechanisms converge, thereby offering unique insights into specific herbs and their compounds. In this study, we obtained data such as correlation scores and protein overlap, which allowed us to prioritize the herbal medicines for MDD treatment by using this approach. Collectively, this approach provides a robust and novel framework for deciphering the intricate pathophysiology of MDD and guiding the discovery of more effective, multi-target therapeutic strategies.

To further investigate our predictions, we conducted a transcriptome analysis using publicly available data from the HERB database and the CMap [28]. Specifically, we retrieved gene expression data for oleanolic acid—a prioritized compound in Campsitis Flos—and compared its differential expression signature. Our analysis revealed a high connectivity score with sertraline, an approved medication for MDD, thereby underscoring the potential applicability of the discovered active compounds in depression treatment. We also identified additional high-similarity compounds, including protease inhibitors, caspase activators, and HDAC/kinase inhibitors, that modulate inflammatory, apoptotic, and stress-response pathways. Enrichment analysis also highlighted its related biological functions, including hsp90 protein binding, aligning with previous findings that highlight its role in stress and glucocorticoid regulation and its reported dysregulation in MDD [29]. These transcriptome-wide findings provide additional evidence supporting the potential of the triterpenic acids in Campsitis Flos as multi-target interventions for depression.

The results of our multiscale network analysis provide information on the putative mechanisms and novel pathways through which the prioritized herbs may exert therapeutic effects against MDD. Each herb’s active compounds connect with multiple MDD-related targets, underscoring the potential for simultaneous modulation of diverse biological pathways. *Ephedrae Herba*’s active compounds, such as pseudoephedrine, were shown to influence proteins involved in oxidative stress (e.g., NOS2, TP53, NFE2L2), transcriptional regulation (e.g., HDAC4, ATF6), and inflammatory signaling (e.g., TNF, NFKBIA). By modulating these processes, *Ephedrae Herba* may help rebalance neurotransmitter systems, endocrine responses, and immune mechanisms often dysregulated in MDD. Additionally, tetramethylpyrazine was found to interact with several tubulin family proteins (e.g., TUBB, TBC1D3, TUBB2B, TUBB3, TUBA1A), which play crucial roles in cytoskeletal organization, intracellular transport, and neuronal plasticity. By modulating these processes, Ephedrae Herba may contribute to the maintenance and restoration of neuronal structural and functional integrity, thereby potentially mitigating dysregulations in neurotransmission, stress responses, and immune mechanisms commonly observed in MDD. Meanwhile, *Glehniae radix*’s main compounds (including isoimperatorin, xanthotoxin, falcarindiol, and psoralen) engage in pathways regulating neuroinflammation, stress responses, and hormone metabolism. Specifically, key targets such as DRD1, NOS2, ESR1, and MAOA indicate the herb’s potential to influence oxidation–reduction processes—thus mitigating oxidative stress—and steroid metabolic processes, potentially normalizing neuroendocrine function [30,31,32,33,34]. These pathways intersect with NF-κB and MAPK signaling, both of which are associated with the production of pro-inflammatory cytokines and stress-related mediators in MDD [35,36,37,38]. Although *Euryales Semen* and *Campsitis Flos* exhibit distinct target profiles, all four herbs illustrate the advantage of a multi-target therapeutic approach in addressing MDD’s multifactorial pathophysiology. In contrast to single-target strategies, each herb’s set of active compounds may collectively modulate neurotransmission, inflammatory cascades, endocrine regulation n, and cellular stress responses. These combined influences could help restore homeostatic balance across multiple pathways implicated in MDD symptomatology. Overall, these mechanistic insights emphasize the importance of network-based methodologies in uncovering how complex mixtures—such as herbal medicines—can act through multiple, interconnected targets.

Although our results suggest promising roles for the prioritized herbs and their active compounds in MDD, several limitations must be acknowledged. First, while *Ephedrae herba* achieved a high correlation score, two of its principal compounds—ephedrine and pseudoephedrine—are generally considered unsuitable for MDD patients due to potential adverse effects. This potential discrepancy illustrates the gap between computational predictions and real-world clinical applicability. Nonetheless, other components within *Ephedrae Herba* (e.g., epicatechin, tyrosine) and the remaining prioritized herbs demonstrated favorable correlation profiles and network connections, suggesting that our predictive framework is capable of identifying compounds with therapeutic potential. Furthermore, this study relies on publicly available databases and computational algorithms, which may introduce biases or inaccuracies in compound–target associations. However, we minimized such biases by focusing on experimentally validated compound–target interactions from resources like DrugBank, TTD, and STITCH. Also, the multiscale network approach employed in this study carries certain limitations. In particular, although the biased random walk with restart algorithm excels at integrating multi-layer network information and highlighting multi-target relationships [11], it does not fully capture potential synergistic or antagonistic interactions among different compounds. Hence, future in vitro and in vivo investigations would be an intriguing next step, offering essential validation of our computational predictions and laying the groundwork for safer, more effective multi-target approaches to MDD treatment. For example, initial cell-based assays could be employed to confirm the ability of prioritized compounds to modulate key MDD-associated signaling pathways, followed by animal model studies to evaluate behavioral outcomes, toxicity profiles, and pharmacokinetics. Overall, by systematically identifying candidate herbs and compounds through a robust, multi-target network framework, this study provides a foundation for the development of natural product-based interventions.

## 4. Materials and Methods

### 4.1. Herb–Component–Target Network Construction

Information on herbs and their constituent components was obtained from the OASIS traditional medicine database (https://oasis.kiom.re.kr/index.jsp, accessed on 29 January 2025), managed by the Korean Institute of Oriental Medicine (KIOM). The OASIS platform consolidates data on potentially active compounds derived from herbal medicines, identified through techniques as HPLC or UPLC, and verified by pharmacological and traditional medicine experts. From this resource, we gathered 12,871 herb–component associations involving 420 herbs and 4786 components, each annotated with a unique PubChem CID. These component data served as the foundational input for our network analysis. We then collected experimentally validated component–target interaction information from databases—including DrugBank 5.0, the Therapeutic Target Database (TTD 2.0), and the Search Tool for Interactions of Chemicals (STITCH, version 5)—which offer comprehensive details on known and potential targets, associated diseases, and biological processes [39,40,41]. To ensure accurate target matching, SynGO (version 1.2) was employed to align gene symbols and UniProt IDs with Entrez Gene IDs [42].

Following data collection, a network was constructed to represent the relationships among herbs, components, and protein targets. In this network, nodes corresponded to each of these three entity types, while edges denoted herb–component and component–target interactions. All edges were unweighted and undirected, indicating the mere presence of an interaction rather than its direction or strength. Components identified by PubChem CID were cross-referenced with the component–target datasets, and herbs containing fewer than three target-linked components were excluded to maintain robust analytical quality. This threshold was chosen because herbs with at least three active components are more likely to exhibit substantive pharmacological effects and yield reliable interaction data. The resulting network enabled the visualization and analysis of interconnections among herbs, their constituents, and protein targets. Within this framework, a simple pathway count was calculated for each herb, accommodating instances where multiple components acted on a single target. This facilitated the selection of the top 50 targets, each evaluated for relative importance, and provided a solid foundation for subsequent investigation.

### 4.2. Enrichment Analysis

Enrichment analysis was performed using the GSEApy module (version 1.1.3) in a Python 3.7 environment, leveraging the Enrichr platform (http://amp.pharm.mssm.edu/Enrichr/, accessed on 23 January 2025) [43,44]. Enrichr incorporates multiple gene-set libraries, such as Gene Ontology (GO) and the Kyoto Encyclopedia of Genes and Genomes (KEGG) [45,46], to conduct comprehensive enrichment assessments. Adjusted *p*-values, z-scores, and combined scores were calculated to identify the signaling pathways and biological functions most relevant to the herbal component targets. Notably, the combined score—derived by multiplying the log-transformed *p*-value by the z-score—served as a reliable metric for assessing the potential influence of these herbal constituents on specific biological pathways. All signaling pathways uncovered through enrichment analysis were included, except those explicitly tied to known diseases.

### 4.3. Disease-Related Targets

The MDD-related protein dataset curated by Ruiz et al. was used to identify proteins implicated in MDD [47]. This dataset was obtained from DisGeNet 2019 (https://www.disgenet.org/, accessed on 23 January 2025)—a comprehensive resource that meticulously maps disease–gene associations to enhance research reliability [48]. We focused on expert-curated disease–gene associations specifically labeled “Major Depressive Disorder,” ensuring direct relevance to our research objectives. The curated dataset draws from authoritative sources such as UniProt, the Comparative Toxicogenomics Database, Orphanet, the Clinical Genome Resource, Genomics England PanelApp, Cancer Genome Interpreter, and the Psychiatric Disorders Gene Association Network. We excluded any associations based on animal model homology, derived solely from computational literature mining, or classified as therapeutic. The resulting disease–protein interaction network served as the foundation for validating MDD-related protein information in our analysis.

### 4.4. Multiscale Network Analysis for Predicting Disease Associations

A multiscale network was retried from the methodology of Ruiz et al. [47], integrating three distinct interaction types: protein–protein, protein–biological function, and biological function–function. They gathered human protein–protein interaction data from BioGRID, the Database of Interacting Proteins (DIP), and the Human Reference Protein Interactome Mapping Project (HuRI), encompassing 387,626 physical interactions among 17,660 proteins. We obtained protein–biological function interactions from the human Gene Ontology database, yielding 34,777 experimentally validated associations between 7993 proteins and 6387 biological functions. Finally, we organized biological function–function interactions into a densely connected hierarchical structure, comprising 22,545 associations across 9798 functions. The full dataset used to construct this network is publicly available on GitHub at https://github.com/snap-stanford/multiscale-interactome (accessed on 8 July 2024).

Using the constructed network, diffusion profiles were simulated to evaluate how potential targets might influence MDD-associated proteins. A biased random walk with restart algorithm was employed to quantify the propagation of these targets toward MDD-related proteins. Correlation scores between the herb–component and disease-specific profiles were then calculated, enabling the identification of prioritized herbs and components with potential therapeutic value. To determine the primary mechanisms underlying each component–disease pair, diffusion profiles were analyzed, and the top k proteins or biological functions most influenced by either the component or the disease were selected. A network was subsequently constructed from these entities to underscore their significance, and any component targets not linked to disease-associated proteins or biological functions were excluded. The highest-ranking entity in each diffusion profile was deemed most critical for treatment due to its substantial impact. The value of k was set to 20 to capture a sufficiently large fraction of nodes exhibiting high visitation frequencies. Further details on the diffusion profile calculation—including mathematical formulations, iterative procedures, and the rationale for selecting k—are provided in Ruiz et al. [47].

### 4.5. Transcriptome Analysis

A transcriptome-based validation was performed using data from the HERB database (http://herb.ac.cn/, accessed on 14 February 2025) [28]. The HERB database integrates experimental evidence—including curated connections to the Connectivity Map (CMap)—enabling cross-referencing of gene expression profiles associated with various bioactive compounds. From the HERB database, CMap-based similarity scores were also retrieved to determine how closely each compound’s DEG profile aligned with those of other bioactive substances, suggesting overlapping mechanisms of action. Compounds with a summary score ≥80 were classified as highly similar, and their functional activities were cross-referenced with MDD-related literature to identify key pathways. Transcriptome profiles for the targeted compounds were also obtained, and differentially expressed genes (DEGs) were identified following standard preprocessing steps, including background correction and normalization. Genes meeting the criteria of |log2 (fold-change)| ≥ 1.0 and an adjusted *p*-value < 0.05 were considered significant. Gene Ontology (GO) enrichment analysis was performed using clusterProfiler to characterize the biological pathways potentially influenced.

## 5. Conclusions

In conclusion, we proposed a multiscale network approach to systematically identify and prioritize herbs and their active compounds that could be beneficial for MDD. Our analyses revealed that *Ephedrae herba*, *Glehniae radix*, *Euryales semen*, and *Campsitis flos* each engage in multiple biological pathways—including oxidative stress regulation, neuroinflammation, and hormone metabolism—highlighting the value of multi-target strategies in addressing the complex etiology of MDD. By integrating various layers of molecular data, we uncovered potential mechanisms that a single-target approach may overlook. These findings not only strengthen our understanding of how individual herbs may modulate depression-relevant pathways but also support the broader application of network-based methodologies in psychiatric drug discovery.

## Figures and Tables

**Table 1:** Prioritized herbs for MDD.

Herb Name (Latin)	Correlation Score	Overlap (*p*-Value)	Enrichment	References (PMID)
*Bufo*	0.0131	11/51 (7.58 × 10^−6^)	5.03	33828600 [12]
*Ephedrae herba*	0.0122	10/50 (3.99 × 10^−5^)	4.66	-
*Glehniae radix*	0.0108	12/30 (1.31 × 10^−9^)	9.32	-
*Crotonis semen*	0.0106	8/50 (1.15 × 10^−3^)	3.73	32316571 [13]
*Euryales semen*	0.0104	8/31 (1.15 × 10^−3^)	3.73	-
*Acori graminei rhizoma*	0.0104	11/47 (3.23 × 10^−6^)	5.45	27685847, 33316383, 37007031 [14,15,16]
*Campsitis flos*	0.0103	11/50 (6.18 × 10^−6^)	5.13	-
*Scorpio*	0.0102	6/50 (1.92 × 10^−2^)	2.80	-
*Selaginellae herba*	0.0102	8/50 (1.15 × 10^−3^)	3.73	-
*Kochiae fructus*	0.0101	14/50 (1.18 × 10^−8^)	6.52	-

**Table 2:** KEGG signaling pathway enrichment analysis of key protein targets.

Term	Overlap	Adjusted *p*-Value	Combined Score	Targets
Neuroactive ligand–receptor interaction	33/341	8.54 × 10^−45^	9704.22	LPAR1; ADRB1; ADRA1D; ADRB2; ADRA1B; ADRA1A; LTB4R; EDNRA; CCKAR; EDNRB; CNR2; CNR1; ADORA3; ADORA1; BDKRB1; DRD1; DRD2; DRD3; DRD4; DRD5; AVPR1B; AVPR2; AVPR1A; ADRA2C; ADRA2B; ADRA2A; ADORA2A; ADRB3; CCKBR; ADORA2B; AGTR1; APLNR; AGTR2
Calcium signaling pathway	19/240	2.97 × 10^−23^	2443.31	AVPR1B; NOS2; ADRB1; ADRA1D; ADRB2; AVPR1A; ADRA1B; ADRA1A; EDNRA; CCKAR; EDNRB; ADORA2A; ADRB3; CCKBR; ADORA2B; AGTR1; BDKRB1; DRD1; DRD5
cGMP-PKG signaling pathway	16/167	6.02 × 10^−21^	2503.92	BAD; ADRB1; ADRA1D; ADRB2; ADRA1B; ADRA2C; ADRA1A; ADRA2B; ADRA2A; EDNRA; EDNRB; ADRB3; ADORA3; AGTR1; ADORA1; MAPK1
Vascular smooth muscle contraction	10/133	2.72 × 10^−12^	953.66	EDNRA; ADORA2A; AVPR1B; ADORA2B; AGTR1; MAPK1; ADRA1D; AVPR1A; ADRA1B; ADRA1A
cAMP signaling pathway	11/216	1.43 × 10^−11^	601.21	EDNRA; ADORA2A; BAD; ADORA1; MAPK1; ADRB1; ADRB2; DRD1; DRD2; NFKB1; DRD5
IL-17 signaling pathway	8/94	1.78 × 10^−10^	882.26	IL6; IL1B; CASP3; MAPK1; MAPK14; PTGS2; TNF; NFKB1
Pathways of neurodegeneration	13/475	3.80 × 10^−10^	283.13	NOS2; BAD; MAPK14; PTGS2; TNF; MTOR; NFKB1; CASP9; IL6; IL1B; CASP3; BAX; MAPK1
cGMP-PKG signaling pathway	16/167	6.02 × 10^−21^	2503.92	BAD; ADRB1; ADRA1D; ADRB2; ADRA1B; ADRA2C; ADRA1A; ADRA2B; ADRA2A; EDNRA; EDNRB; ADRB3; ADORA3; AGTR1; ADORA1; MAPK1
Vascular smooth muscle contraction	10/133	2.72 × 10^−12^	953.66	EDNRA; ADORA2A; AVPR1B; ADORA2B; AGTR1; MAPK1; ADRA1D; AVPR1A; ADRA1B; ADRA1A
cAMP signaling pathway	11/216	1.43 × 10^−11^	601.21	EDNRA; ADORA2A; BAD; ADORA1; MAPK1; ADRB1; ADRB2; DRD1; DRD2; NFKB1; DRD5

## Data Availability

The data supporting the findings of this study are included within the manuscript.

## Supplementary Materials

The supporting information can be downloaded at https://www.mdpi.com/article/10.3390/ijms26052162/s1.
